# Genesis of interictal spikes in the CA1: a computational investigation

**DOI:** 10.1186/1471-2202-13-S1-P30

**Published:** 2012-07-16

**Authors:** RG Shivakeshavan, Roxana A Stefanescu, Pramod P Khargonekar, Paul R Carney, Sachin S Talathi

**Affiliations:** 1 Department of Biomedical Engineering, University of Florida, Gainesville, FL 32610, USA; 2 Department of Pediatrics, University of Florida, Gainesville, FL 32610, USA; 3 Department of Electrical and Computer Engineering, University of Florida, Gainesville, FL 32610, USA

## 

Interictal spikes (IIS) refer to abnormal synchronized neuronal discharges, which are hallmarks of epilepsy and can be confirmed through electroencephalography (EEG) [1]. In vitro analysis of resected temporal lobe tissue from patients with refractory temporal lobe epilepsy of hippocampal origin has revealed the presence of IIS in the CA1 subfield. IIS are abolished when the Schaffer collateral (SC) input from the CA3 subfield is cut [2]. The CA1 undergoes morphological changes including cell death and the emergence of recurrent connections between excitatory neurons following brain injury that triggers epileptic seizures [3].

A question then arises: How are the morphological network changes and input variability through SC related to the CA1’s ability to generate IIS? To address this issue, we develop a computational model of the CA1 network. We characterize the morphological changes within the CA1 network by changing the percentage of recurrent synaptic connections (average number of incoming synapses onto CA1 pyramidal cells). Input variability is studied in two ways: (i) By changing the fraction of CA1 pyramidal cells that receive input from SC, and (ii) by changing the synchronization of incoming input by varying the temporal window in which SC input arrives. The CA1 computational network is comprised of 280 neurons of which 80% are pyramidal (excitatory) and 20% are inhibitory interneurons. Each neuron is modeled using the Hodgkin-Huxley framework of conductance based point neuron models.

Since pathological burst firing of pyramidal neurons, referred to as the paroxysmal depolarization shift (PDS) are known as the cellular correlates for IIS, we begin by identifying a typical temporal profile of a cellular PDS event that contributes to an observed IIS. We begin with experimental data on IIS recorded from the CA1 subfield of an *in vivo* self-sustaining status electrical status epilepticus animal model of chronic limbic epilepsy [4]. We then generate template IIS events using a subset of artificial PDS constructs. By matching the temporal profile of the template IIS with the experimental IIS, we identify the characteristics for PDS constructs that can generate experimentally observable IIS. We then use this information to tune the synaptic parameters of a minimal network of a pyramidal cell coupled with interneurons that is capable of generating a PDS event that matches the temporal profile of the artificial PDS construct in response to stimulation from external synaptic input. The minimal model parameters are then incorporated into a detailed CA1 network model to study conditions under which experimentally observable IIS are produced.

We find that the CA1 network with low recurrent connectivity, mimicking the topology of a normal brain, has a very low probability of producing an IIS except when a large fraction of CA1 neurons (>80%) receives a quasi-synchronous barrage of input (events occurring within a temporal window of 20 ms) via SC. However, as we increase recurrent connectivity of CA1 (>40%) we find that an IIS can be evoked in the CA1 network even in the presence of low synchrony SC input (>80 ms temporal window) and a low fraction of SC input to CA1 pyramidal cells (>30%). For sufficiently high recurrent connections (40%), the model produces a sequence of IIS in response to sparse asynchronous SC input. These results indicate that as the CA1 becomes increasingly excitable resulting from recurrent excitatory connections, the ability to produce IIS increases with less dependence on input variability. This in turn, suggests that the CA1’s susceptibility to produce IIS increases following brain injury, a finding that has been reported in earlier experimental studies [5].
